# A plan forward: an assessment of workforce concerns and supportive initiatives for dermatologist parents and caregivers

**DOI:** 10.1097/JW9.0000000000000229

**Published:** 2025-10-17

**Authors:** Shannon T. Nugent, John Miller, Rosie Balk, Jeffrey Miller, Sherry Yang, Elizabeth Jones

**Affiliations:** a Department of Dermatology, Hospital of the University of Pennsylvania, Philadelphia, Pennsylvania; b Department of Dermatology and Cutaneous Biology, Thomas Jefferson University Hospital, Philadelphia, Pennsylvania; c American Academy of Dermatology Association, Rosemont, Illinois

**Keywords:** caregivers, childcare, parents

**What is known about this subject in regard to women and their families?**

- The 2024 Surgeon General Advisory highlighted worsening parental stress as a critical public health issue, urging employers to better support the well-being of parents and caregivers.
- Previous studies show that childcare stress correlates with increased anxiety, burnout, and likelihood to reduce clinical hours or leave clinical practice.
- This relationship has not been well characterized among board-certified dermatologists.

**What is new from this article as messages for women and their families?**

- Our study is the first of its kind to characterize how childcare responsibilities impact the careers of practicing board-certified dermatologists through a survey distributed to members of the American Academy of Dermatology, Women’s Dermatologic Society, and Association of Professors of Dermatology.
- Our study highlights the significant demands that parent dermatologists face and highlights solutions to improve support and foster a sustainable and diverse workforce.

## Introduction

The 2024 Surgeon General Advisory highlighted worsening parental stress as a critical public health issue, urging employers to better support the well-being of parents and caregivers. Among healthcare workers, childcare stress correlates with increased anxiety, burnout, and likelihood to reduce clinical hours or leave clinical practice.^1^ This study aims to quantify childcare responsibilities, identify the impact on career development, and explore potential solutions identified by board-certified dermatologists.

## Methods

This project was approved by the Institutional Review Board of Thomas Jefferson University. From October 24 to December 18, 2023, members of the American Academy of Dermatology (AAD), Women’s Dermatologic Society, and Association of Professors of Dermatology were recruited to participate in a one-time anonymous email survey sent to members through organizations’ email list (eSurvey included in supplementary materials https://links.lww.com/IJWD/A76). Eligible respondents were practicing board-certified dermatologists caring for at least one minor. Questions were influenced by prior studies.^1–3^ Data collection and analysis were performed by the AAD. Descriptive statistics were used to calculate demographics and response frequency. Pearson’s chi-square test evaluated the relationship between survey responses for all male versus female responses with a false discovery rate adjustment applied to control for multiple comparisons. Statistical analysis was performed with DisplayR software.

## Results

Of the 4,007 board-certified dermatologists who received the survey, an estimated 1,600 were expected to meet the inclusion criteria based on internal AAD membership demographic data. A total of 553 responded, yielding a response rate of 35% among expected eligible participants. Most respondents (87%) completed the survey in its entirety. The average age of participants was 42.2 years, and 80% of respondents identified as female. Participants reported having 2.3 children on average. Children’s ages ranged from 0 to 2 years (36%), 3 to 5 years (34%), 6 to 12 years (50%), and 13 to 18 years (33%).

Table 1 illustrates the types of childcare utilized by dermatologists each workweek. The most common arrangements included nanny/babysitter (43%) and spouse (42%). Women were significantly more likely to rely on a nanny/babysitter or themselves, while men depended more on spouses. Women also reported higher childcare costs than men ($2,334 vs $1,577).

**Table 1:** Types of childcare utilized by dermatologists and time allocated per workweek by gender

Type of childcare	Total (n = 476)	Women (n = 379)	Men (n = 97)	*P* value
Nanny/babysitter	43% (206)	47% (179)	28% (27)	<.001
Spouse	42% (200)	36% (136)	66% (64)	<.001
Primary school	39% (185)	39% (146)	40% (39)	1.00
Self (dermatologist)	35% (185)	38% (143)	26% (25)	.12
Extended family	26% (122)	27% (104)	19% (18)	.32
Preschool	22% (104)	23% (104)	16% (16)	.67
External daycare	18% (84%)	19% (72)	12% (12)	.56
Time allocated (hours/week)	Total (n = 476)	Women (n = 379)	Men (n = 97)	*P* value
Total average hours allocated per week	62.6	63.0	61.2	1.00
Primary school	13.2	12.7	15.0	1.00
Nanny/babysitter	12.2	13.7	6.5	<.001
Spouse	9.9	6.3	23.9	<.001
Self (dermatologist)	6.9	7.6	3.9	.014
Preschool	6.4	7.0	4.0	.018
External daycare	6.1	6.7	3.6	.019
Extended family	4.3	4.9	2.1	.012
Average total cost of childcare per month	Total (n = 472)	Women (n = 375)	Men (n = 97)	*P* value
Amount	$2179	$2334	$1577	.002

Table 2 highlights work-life balance and current childcare practices. Approximately 37% of respondents reported burnout at least weekly, and 33% were very or somewhat dissatisfied with work-life balance. Dissatisfaction with employer parental support was reported by 26% of respondents. Key improvement priorities included paternal leave (42%), subsidized childcare costs (41%), paid time off/vacation (39%), and scheduling accommodations (38%). Women were significantly more likely to shift to part-time status or switch practice location compared to men.

**Table 2:** Evaluation of satisfaction with current childcare and identification of solutions preferred by respondents

Question	%, (n=total)
I feel burnout from my work	n = 484
Every day	9.5 (46)
A few times per week	17.6 (85)
Once a week	9.5 (46)
A few times per month	22.7 (110)
Once a month	16.9 (82)
A few times per year or less	19.0 (92)
Never	4.8 (23)
How satisfied are you with work-life balance	n = 480
Very dissatisfied	6.5 (31)
Somewhat dissatisfied	26.5 (127)
Neutral	13.8 (174)
Somewhat satisfied	36.3 (174)
Very satisfied	17.1 (82)
How satisfied are you with your current childcare plan	n = 470
Very dissatisfied	1.5 (7)
Somewhat dissatisfied	9.8 (46)
Neutral	15.1 (71)
Somewhat satisfied	38.7 (182)
Very satisfied	34.9 (164)
How satisfied are you with the time that you’re able to spend with your children	n = 482
Very dissatisfied	8.9 (43)
Somewhat dissatisfied	25.9 (125)
Neutral	8.9 (43)
Somewhat satisfied	38.2 (184)
Very satisfied	18.0 (87)
How satisfied are you with the support that you receive as a parent from your employer	n = 418
Very dissatisfied	10.3 (43)
Somewhat dissatisfied	15.3 (64)
Neutral	26.8 (112)
Somewhat satisfied	25.8 (108)
Very satisfied	21.8 (91)
Due to childcare demands I have…	n = 463
Reduced clinical hours	47 (207)
Shifted to part-time status	36 (166)*
Switched practice location	19 (88)**
I would have been able to focus on my job duties more if my practice had provided better support in the form of…	n = 471
Improved maternity/paternity leave	42 (197)
Subsidized childcare costs	41 (194)
Increased vacation/paid time off	39 (184)
Improved accommodation of scheduling changes	38 (180)
Employer-sponsored nanny/babysitting services	34 (159)
Adjustment for incentive pay productivity models of leave/breastfeeding	33 (156)
Flexible full-time schedules	30 (142)
Career advancement and promotion guidance for parents	30 (141)
Flexible part-time schedules	23 (107)
Adjustment in promotion criteria for leave	15 (71)

* Statistically significant difference between men and women respondents, *P* < .001.

** Statistically significant difference between men and women respondents, *P* < .05.

Lastly, childcare responsibilities influenced career decisions, with nearly half of respondents reducing clinical hours and one-third transitioning to part-time roles. Women were significantly more likely to shift to part-time work or change job location. Career advancement was also affected, with most respondents noting reduced ability to attend conferences, network, or pursue leadership roles (eTable 1 https://links.lww.com/IJWD/A77).

## Discussion

Childcare demands significantly impact dermatologists’ professional lives. Dermatologists often relied on paid childcare, with monthly expenses averaging $2,165. Women faced significantly higher monetary contributions, self-dedicated childcare hours, and likelihood to transition to part-time status compared with men. The rate of burnout in our study aligns with national trends associating childcare responsibilities, particularly among women, with increased burnout across all medical specialties.^4,5^ Given that gender trends in dermatology trainees have shifted to a 3:2 female-to-male ratio based on internal AAD membership demographic data, disparities in childcare responsibilities may affect workforce retention. Improved parental leave and paid time off, subsidized childcare costs, employer-sponsored childcare, and adjusted incentive models with improved accommodation of parental scheduling changes were identified as possible solutions to foster a sustainable and diverse dermatology workforce.

Limitations of this study include the low overall response rate, variable response rate per question, and potential for selection and recall bias, which may influence the generalizability of our findings. Nevertheless, the results highlight the impact of parental responsibilities on dermatologists’ career development and potential directions for future change.

## Conflicts of interest

None.

## Funding

None.

## Study approval

Reviewed and approved by the Thomas Jefferson University IRB.

## Author contribution

Data management and analysis at the AAD was led by RB, Cindy Nelson, and JM. We thank the AAD, APD, and WDS for providing access to their membership and facilitating survey implementation. None of these contributors was compensated for their work on this article.

## Supplementary data

Supplementary material associated with this article can be found at https://links.lww.com/IJWD/A76 and https://links.lww.com/IJWD/A77.

## Supplementary Material


