# Supplementary material for: A plan forward: an assessment of workforce concerns and supportive initiatives for dermatologist parents and caregivers
Source: Int J Womens Dermatol. 2025 Oct 17;11(3):e229. doi: 10.1097/JW9.0000000000000229 (PMC12537157; doi:10.1097/JW9.0000000000000229)
Supplement: Supplementary file 1 [file jw9-11-e229-s001.pdf]

Childcare survey

Start of Block: Default Question Block

Q0 This survey covers some of your experiences as a working dermatologist and the parent/guardian of at least one minor child. Thanks in advance for participating.

Page Break

Q1 Are you a parent or legal guardian of a minor?

- Yes (1)
- No (2)

Skip To: End of Survey If Are you a parent or legal guardian of a minor? = 2

Page Break

Display This Question:

If AADCountryArea Is Empty

RES SCREENER Which of the following best describes you?

- ○ Practicing dermatologist (1)
- ○ Resident (4)
- ○ Fellowship (5)
- ○ Retired dermatologist (6)
- ○ Other (specify) (7) ____

Page Break

Q2 How many children do you have?

- ○ 1 (1)
- ○ 2 (2)
- ○ 3 (3)
- ○ 4 (4)
- ○ 5 (5)
- ○ 6 (6)
- ○ 7 (7)
- ○ 8 (8)
- ○ 9 (9)
- ○ 10 (10)
- ○ More than 10 (11)

Page Break

Display This Question:

If How many children do you have? = 11

Q2a Please answer the following question for your 10 youngest children.

Page Break

Q2b What are the ages of your children?

Display This Choice:

If How many children do you have? = 1

Or How many children do you have? = 2

Or How many children do you have? = 3

Or How many children do you have? = 4

Or How many children do you have? = 5

Or How many children do you have? = 6

Or How many children do you have? = 7

Or How many children do you have? = 8

Or How many children do you have? = 9

Or How many children do you have? = 10

Or How many children do you have? = 11

Display This Choice:

If How many children do you have? = 2

Or How many children do you have? = 3

Or How many children do you have? = 4

Or How many children do you have? = 5

Or How many children do you have? = 6

Or How many children do you have? = 7

Or How many children do you have? = 8

Or How many children do you have? = 9

Or How many children do you have? = 10

Or How many children do you have? = 11

Display This Choice:

If How many children do you have? = 3

Or How many children do you have? = 4

Or How many children do you have? = 5

Or How many children do you have? = 6

Or How many children do you have? = 7

Or How many children do you have? = 8

Or How many children do you have? = 9

Or How many children do you have? = 10

Or How many children do you have? = 11

Display This Choice:

If How many children do you have? = 4

Or How many children do you have? = 5

Or How many children do you have? = 6

Or How many children do you have? = 7

Or How many children do you have? = 8

Or How many children do you have? = 9

Or How many children do you have? = 10

Or How many children do you have? = 11

Display This Choice:

If How many children do you have? = 5

Or How many children do you have? = 6

Or How many children do you have? = 7

Or How many children do you have? = 8

Or How many children do you have? = 9

Or How many children do you have? = 10

Or How many children do you have? = 11

Display This Choice:

If How many children do you have? = 6

Or How many children do you have? = 7

Or How many children do you have? = 8

Or How many children do you have? = 9

Or How many children do you have? = 10

Or How many children do you have? = 11

Display This Choice:

If How many children do you have? = 7

Or How many children do you have? = 8

Or How many children do you have? = 9

Or How many children do you have? = 10

Or How many children do you have? = 11

Display This Choice:

If How many children do you have? = 8

Or How many children do you have? = 9

Or How many children do you have? = 10

Or How many children do you have? = 11

Display This Choice:

If How many children do you have? = 9

Or How many children do you have? = 10

Or How many children do you have? = 11

Display This Choice:

If How many children do you have? = 10

Or How many children do you have? = 11

		1-2 years (2)	3-5 years (3)	6-12 years (4)	13-18 years (5)	>18 years (6)

Display This Choice: *If How many children do you have? = 1* *Or How many children do you have? = 2* *Or How many children do you have? = 3* *Or How many children do you have? = 4* *Or How many children do you have? = 5* *Or How many children do you have? = 6* *Or How many children do you have? = 7* *Or How many children do you have? = 8* *Or How many children do you have? = 9* *Or How many*	○	○	○	○	○	○

children do you have? = 10 *Or How many children do you have? = 11*
Child 1 (1)

Display This Choice:<br>*If How many children do you have? = 2*<br>*Or How many children do you have? = 3*<br>*Or How many children do you have? = 4*<br>*Or How many children do you have? = 5*<br>*Or How many children do you have? = 6*<br>*Or How many children do you have? = 7*<br>*Or How many children do you have? = 8*<br>*Or How many children do you have? = 9*<br>*Or How many children do you have? = 10*<br>*Or How many*	○	○	○	○	○	○

children do you have? = 11
Child 2 (2)

Display This Choice:<br>*If How many children do you have? = 3*<br>*Or How many children do you have? = 4*<br>*Or How many children do you have? = 5*<br>*Or How many children do you have? = 6*<br>*Or How many children do you have? = 7*<br>*Or How many children do you have? = 8*<br>*Or How many children do you have? = 9*<br>*Or How many children do you have? = 10*<br>*Or How many children do you have? = 11*<br>Child 3 (3)	○	○	○	○	○	○

Display This Choice: *If How many children do you have? = 4* *Or How many children do you have? = 5* *Or How many children do you have? = 6* *Or How many children do you have? = 7* *Or How many children do you have? = 8* *Or How many children do you have? = 9* *Or How many children do you have? = 10* *Or How many children do you have? = 11* Child 4 (4)	○	○	○	○	○	○

Display This Choice:<br>*If How many children do you have? = 5*<br>*Or How many children do you have? = 6*<br>*Or How many children do you have? = 7*<br>*Or How many children do you have? = 8*<br>*Or How many children do you have? = 9*<br>*Or How many children do you have? = 10*<br>*Or How many children do you have? = 11*<br>Child 5 (5)	○	○	○	○	○	○

Display This Choice:<br>*If How many children do you have? = 6*<br>*Or How many children do you have? = 7*<br>*Or How many children do you have? = 8*<br>*Or How many children do you have? = 9*<br>*Or How many children do you have? = 10*<br>*Or How many children do you have? = 11*<br>Child 6 (6)	○	○	○	○	○	○

Display This Choice:<br>*If How many children do you have? = 7*<br>*Or How many children do you have? = 8*<br>*Or How many children do you have? = 9*<br>*Or How many children do you have? = 10*<br>*Or How many children do you have? = 11*<br>Child 7 (7)	○	○	○	○	○	○

Display This Choice: *If How many children do you have? = 8* *Or How many children do you have? = 9* *Or How many children do you have? = 10* *Or How many children do you have? = 11* Child 8 (8)	○	○	○	○	○	○
Display This Choice: *If How many children do you have? = 9* *Or How many children do you have? = 10* *Or How many children do you have? = 11* Child 9 (9)	○	○	○	○	○	○

Display This Choice:<br>*If How many children do you have? = 10*<br>*Or How many children do you have? = 11*<br>Child 10 (10)	○	○	○	○	○	○

Page Break

Q3 What is your gender?

- Woman (1)
- Man (2)
- Non-binary (3)
- Prefer not to answer (4)

Display This Question:

If AADCountryArea Is Empty

DATE OF BIRTH Please enter your date of birth in mm/dd/yyyy format.

- Date of Birth (1) ____

Page Break

Q4 Are you of Hispanic, Latino or Spanish origin?

- ◯ Yes (1)
- ◯ No (2)
- ◯ Prefer not to answer (3)

Page Break

Q5 Which of the following best describes you? (Select all that apply)

- ▢ Asian (1)
- ▢ Biracial (2)
- ▢ Black or African American (3)
- ▢ Middle Eastern (4)
- ▢ Multiracial (5)
- ▢ Native American (6)
- ▢ Pacific Islander (7)
- ▢ White (8)
- ▢ Other (specify) (9) ____
- ▢ ⊗Prefer not to answer (10)

Page Break

Q6 What type of practice is your **primary** practice? (Select one)

- ○ None – I am retired (1)
- ○ Dermatology Group – Please enter the number of dermatologists (2) ____
- ○ Hospital - Academic (3)
- ○ Hospital - Non-Academic (4)
- ○ Multi-specialty Group (5)
- ○ Solo practice (6)
- ○ VA/Military (7)
- ○ Other (specify) (8) ____

Page Break

Display This Question:

If What type of practice is your primary practice? (Select one) = 2

Or What type of practice is your primary practice? (Select one) = 3

Or What type of practice is your primary practice? (Select one) = 4

Or What type of practice is your primary practice? (Select one) = 5

Or What type of practice is your primary practice? (Select one) = 6

Or What type of practice is your primary practice? (Select one) = 7

Or What type of practice is your primary practice? (Select one) = 8

Q7 How is your primary practice **owned**? (Select all that apply)

- ☐ An organization backed by private equity (1)
- ☐ Government (2)
- ☐ Myself/Dermatologist Owners (3)
- ☐ Non-physician owners (4)
- ☐ Part of a Health system/Hospital/Academic Center (5)
- ☐ Physician owned - non-dermatologist (6)
- ☐ Other (specify) (7) ____
- ☐ ⊗Don't know (8)

Page Break

Q8 Are you a dual-income household?

- ○ Yes (1)
- ○ No (2)

Page Break

Display This Question:

If Are you a dual-income household? = 1

Q8a Are you a dual-*physician* household?

- Yes (1)
- No (2)

Page Break

Q9 What is your marital status?

- ○ Married (1)
- ○ Living together, unmarried (2)
- ○ Divorced/Separated (3)
- ○ Single (4)
- ○ Prefer not to answer (5)

Page Break

Q10 What type of childcare do you currently use during a typical school year work week? (Select all that apply)

- ☐ Self (1)
- ☐ Spouse (2)
- ☐ Grandparent/other family (3)
- ☐ Employer provided daycare (4)
- ☐ External daycare (5)
- ☐ Nanny or babysitter (6)
- ☐ Preschool (7)
- ☐ Primary school K-12 (8)
- ☐ Before or after school care (9)
- ☐ Transportation (10)
- ☐ Other (specify) (11) ____
- ☐ ⊗None (12)

Page Break

Display This Question:

If What type of childcare do you currently use during a typical school year work week? (Select all t... = 1

Or What type of childcare do you currently use during a typical school year work week? (Select all t... = 2

Or What type of childcare do you currently use during a typical school year work week? (Select all t... = 3

Or What type of childcare do you currently use during a typical school year work week? (Select all t... = 4

Or What type of childcare do you currently use during a typical school year work week? (Select all t... = 5

Or What type of childcare do you currently use during a typical school year work week? (Select all t... = 6

Or What type of childcare do you currently use during a typical school year work week? (Select all t... = 7

Or What type of childcare do you currently use during a typical school year work week? (Select all t... = 8

Or What type of childcare do you currently use during a typical school year work week? (Select all t... = 9

Or What type of childcare do you currently use during a typical school year work week? (Select all t... = 10

Or What type of childcare do you currently use during a typical school year work week? (Select all t... = 11

Carry Forward Selected Choices from "What type of childcare do you currently use during a typical school year work week? (Select all that apply)"

JS X→

Q10a Please enter the approximate average number of **hours per week**, during a typical school year work week, for each type of childcare you selected, shown below.

- ○ Self (1) ____
- ○ Spouse (2) ____
- ○ Grandparent/other family (3) ____
- ○ Employer provided daycare (4) ____
- ○ External daycare (5) ____
- ○ Nanny or babysitter (6) ____
- ○ Preschool (7) ____
- ○ Primary school K-12 (8) ____
- ○ Before or after school care (9) ____
- ○ Transportation (10) ____
- ○ Other (specify) (11) ____
- ○ ⊗None (12) ____

Page Break

Display This Question:

If What type of childcare do you currently use during a typical school year work week? (Select all t... = 1

Or What type of childcare do you currently use during a typical school year work week? (Select all t... = 2

Or What type of childcare do you currently use during a typical school year work week? (Select all t... = 3

Or What type of childcare do you currently use during a typical school year work week? (Select all t... = 4

Or What type of childcare do you currently use during a typical school year work week? (Select all t... = 5

Or What type of childcare do you currently use during a typical school year work week? (Select all t... = 6

Or What type of childcare do you currently use during a typical school year work week? (Select all t... = 7

Or What type of childcare do you currently use during a typical school year work week? (Select all t... = 8

Or What type of childcare do you currently use during a typical school year work week? (Select all t... = 9

Or What type of childcare do you currently use during a typical school year work week? (Select all t... = 10

Or What type of childcare do you currently use during a typical school year work week? (Select all t... = 11

Q11 If your childcare is not available on a given day, what is your **back-up plan**? (Select all that might apply)

- ▢ Self (1)
- ▢ Spouse (2)
- ▢ Before or after school care (3)
- ▢ Employer provided daycare (4)
- ▢ External daycare (5)
- ▢ Grandparent/other family (6)
- ▢ Nanny or babysitter (7)
- ▢ Preschool (8)
- ▢ Primary school K-12 (9)
- ▢ Take off work (10)
- ▢ Other (specify) (11) ____
- ▢ ⨂No plan (12)
- ▢ ⨂Not applicable to me (13)

Page Break

JS

Q12 What is the **total cost** of your childcare **per month** during the school year? Please enter a number without a “$”, commas, or cents/decimals. Enter "0" if you spend no money on childcare.

◯ total childcare cost per month (1) ____

Page Break

Display This Question:

If If What is the total cost of your childcare per month during the school year? Please enter a number without a “$”, commas, or cents/decimals. Enter "0" if you spend no money on childcare. Text Response Is Not Empty

JS

Q13 How many **days per month** do you pay for child care during the school year? Please enter a number between 0-31.

○ days per month paying for child care (1)

____

Page Break

Q14 Due to childcare demands, have you declined, reduced, or faced a delay in opportunities in the following types of career advancement? (Select all that apply)

- ☐ Leadership and/or administrative roles (1)
- ☐ Teaching (2)
- ☐ Research (3)
- ☐ Conference attendance/planning (4)
- ☐ Committee work (5)
- ☐ Promotion (6)
- ☐ Career-focused socializing/networking (7)
- ☐ Other (specify) (8) ____
- ☐ ⊗No, I have not declined or delayed any career opportunities (9)

Page Break

Q15 Due to parental/caretaking responsibilities, have you done any of the following? (Select all that apply)

- ▢ Reduced clinical patient facing hours; please estimate average hours reduced per week: (1) ____

- ▢ Shifted away from full-time status (2)

- ▢ Switched to a non-clinical position (3)

- ▢ Switched practice location (4)

- ▢ Left medicine for a different career (5)

- ▢ Stopped working/retired (6)

- ▢ Something else (specify) (7) ____

- ▢ ⊗No, I haven't made any of these adjustments (8)

Page Break

Q16 Use the scale provided to describe the likelihood of the following events happening in the next two years **due (specifically) to parental/caretaking responsibilities**:

	Very unlikely (1)	Somewhat unlikely (2)	Neutral (3)	Somewhat likely (4)	Very likely (5)	N/A (-99)
Reducing clinical hours (1)	○	○	○	○	○	○
Leaving current practice (2)	○	○	○	○	○	○

Page Break

Q17 “I feel burnout from my work.”

- Never (1)
- A few times a year or less (2)
- Once a month (3)
- A few times a month (4)
- Once a week (5)
- A few times a week (6)
- Every day (7)

Page Break

Q18 How satisfied are you with each of the following?

	Very dissatisfied (1)	Somewhat dissatisfied (2)	Neutral (3)	Somewhat satisfied (4)	Very satisfied (5)	N/A (6)
Your work-life balance (1)	○	○	○	○	○	○
Your current childcare plan (2)	○	○	○	○	○	○
The time you are able to spend with your children (3)	○	○	○	○	○	○
The support that you receive as a parent from your employer (4)	○	○	○	○	○	○

Page Break

Q19 *Complete the following statement by selecting all that apply from the list below:*
I felt I would have been able to focus on my job duties more if my practice had provided better support in the form of...

- ▢ Flexible full-time schedules (1)
- ▢ Flexible part-time schedules (2)
- ▢ Improved accommodation of scheduling changes (3)
- ▢ Employer-sponsored childcare daycare (4)
- ▢ Employer-sponsored nanny/babysitting services (5)
- ▢ Increased vacation/paid time off (6)
- ▢ Improved maternity/paternity leave (7)
- ▢ Adjustment in promotion criteria for leave (8)
- ▢ Adjustment for incentive pay productivity models for leave/breastfeeding (9)
- ▢ Career advancement and promotion guidance for parents (10)
- ▢ Subsidizing childcare costs (11)
- ▢ Other (specify) (12) ____
- ▢ ⊗Not applicable to me (13)

Page Break

Q20 What else would you like to tell us about being both a dermatologist and a parent?

____

____

____

____

____

End of Block: Default Question Block
