# Supplementary material for: A plan forward: an assessment of workforce concerns and supportive initiatives for dermatologist parents and caregivers
Source: Int J Womens Dermatol. 2025 Oct 17;11(3):e229. doi: 10.1097/JW9.0000000000000229 (PMC12537157; doi:10.1097/JW9.0000000000000229)
Supplement: Supplementary file 2 [file jw9-11-e229-s002.pdf]

eTable 1. Impact of Childcare Responsibilities on Dermatologist Career Decisions, Job Satisfaction, and Identified Solutions

	**Total (n = 478)**	**Women (n = 379)**	**Men (n = 99)**	**p-value**
Due to childcare demands, I have declined, reduced, or faced delay in the following…				
Attending Conferences	78% (372)	82% (311)	62% (61)	< 0.001
Networking	72% (342)	76% (288)	55% (54)	< 0.001
Leadership Roles	54% (258)	56% (213)	45% (45)	0.337
Committee Work	41% (198)	42% (160)	38% (38)	1.00
Research	39% (186)	40% (151)	38% (38)	1.00
Teaching	37% (178)	39% (146)	32% (32)	1.00
Promotion	22% (106)	22% (85)	21% (21)	1.00
No, I have not declined or delayed any career opportunities	13% (64)	9% (33)	31% (31)	< 0.001
	**Total (n = 463)**	**Women (n = 369)**	**Men (n = 94)**	**p-value**
Due to childcare demands, I have…				
Reduced clinical hours	45% (207)	47% (174)	35% (33)	0.144
Shifted to Part-Time	36% (166)	41% (150)	17% (16)	< 0.001
